# The Evaluation of Upper Labial Swelling: An Arteriovenous Malformation

**DOI:** 10.7759/cureus.56533

**Published:** 2024-03-20

**Authors:** Manu Babu, Aishwarya Kothari

**Affiliations:** 1 Otolaryngology-Head and Neck Surgery, Dr. D. Y. Patil Medical College, Hospital & Research Centre, Dr. D. Y. Patil Vidyapeeth, Pune, Pune, IND

**Keywords:** arteriovenous malformations, endovascular embolization, upper lip swelling, lower lip swelling, upper lip reconstruction

## Abstract

Vascular malformations, which include disorders of the lymphatic or vascular systems, can appear in a variety of ways on radiographs, in the radiological department, and histologically. High-flow lesions with direct arteriovenous connections are known as arteriovenous malformations (AVMs). These lesions can cause soft tissue loss and deformity since they are difficult to diagnose early. This case report describes a 75-year-old female who presented with a severe bluish-purple swelling on her top lip. After conducting a thorough investigation, the patient's condition was quickly identified as AVM.

After confirmation by USG Doppler and histological examination, the patient underwent a successful surgical resection that revealed a confined vascular lesion suggestive of AVM. The discussion explores the hemodynamic and embryologic factors that contribute to the formation of AVM, pointing out differences in hemodynamic properties and clinical symptoms. Treatment choices are influenced by the categorization of peripheral AVMs according to clinical standards and angiographic flow characteristics.

## Introduction

A class of congenital anomalies known as vascular malformations is brought on by anomalies in the lymphatic or vascular systems. High-flow lesions known as arteriovenous malformations (AVMs) occur when arteries and veins directly communicate with one another, avoiding the capillary bed [1]. The clinical, radiological, and histological appearances of vascular malformations and malignancies range greatly. Vascular tumors are endothelial neoplasms that exhibit accelerated cell proliferation. Conversely, the aberrant development of vascular components during embryogenesis and fetal life leads to vascular malformations [2]. Many AVMs are misdiagnosed as hemangiomas during infancy only to later cause progressive soft tissue destruction, ulceration, bleeding, and disfigurement [3]. Here, we present a case of a 75-year-old patient who presented with a painful bluish-purple swelling, which upon investigation was promptly diagnosed with AVM and meticulously managed by surgical resection.

## Case presentation

**Figure 1:**
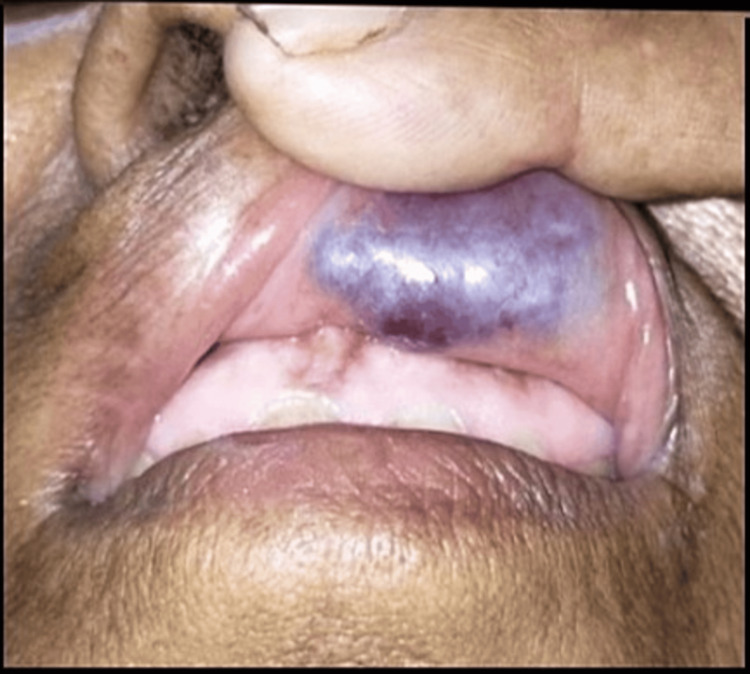
Swelling over the inner aspect of the upper lip.

A 75-year-old female patient presented with swelling over the inner aspect of the upper lip on the left side for 15 days (Figure 1). It was sudden in onset and progressive in nature over 15 days. There was a history of pain over the swelling, which was continuous and dull and did not get relieved by taking medications. She had no complaints of any other similar swelling over any other part of the body. She had no complaints of bleeding or discharge from the swelling. There was no history of a long-standing chronic illness or blood transfusion. Past and family history were not contributory.

On inspection, the external examination of the upper lip showed a swelling of approximately 2×2 cm extending from the columella medially to 1 cm away from the left angle of the mouth laterally. There were no signs of inflammation. The swelling was non-discharging. On everting the upper lip, the mucosal surface of the upper lip showed a swelling of about 2×2 cm extending from the central incisors medially to the left canine laterally. The swelling was bluish red in color. Dilated vessels could be seen over the swelling. There was no discharge from the sinus or bleeding from the swelling. There were no signs of inflammation over the swelling. On palpation of the swelling externally, all the inspection findings were confirmed. The swelling was solitary, ovoid-shaped, and soft to firm in consistency, with a smooth surface and well-defined margins; it was tender; it did not bleed on touch; and there was no local rise in temperature. The rest of the otorhinolaryngological, head and neck, and general examinations were normal.

**Figure 2:**
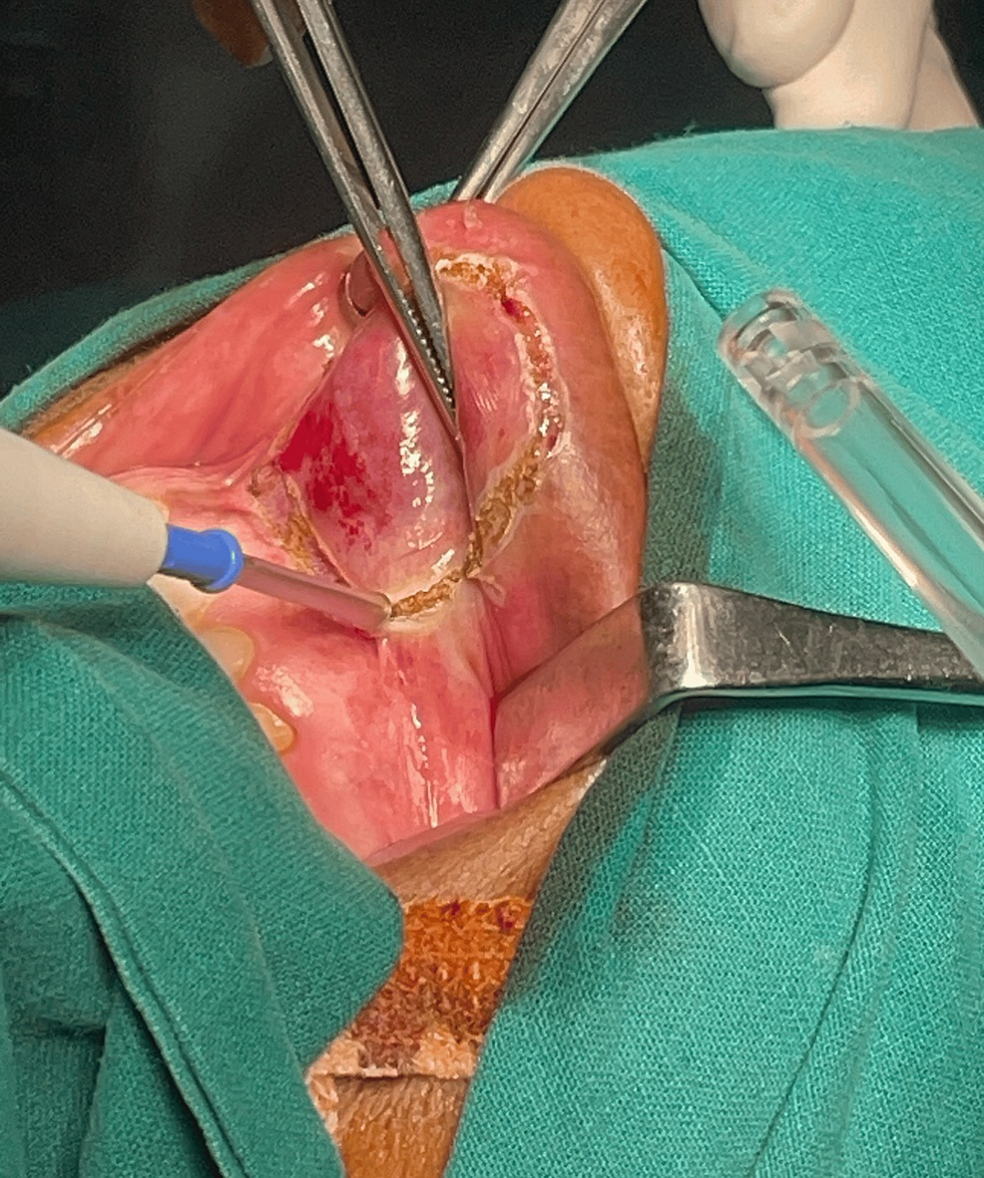
The mass was dissected and excised from its base using cautery.

**Figure 3:**
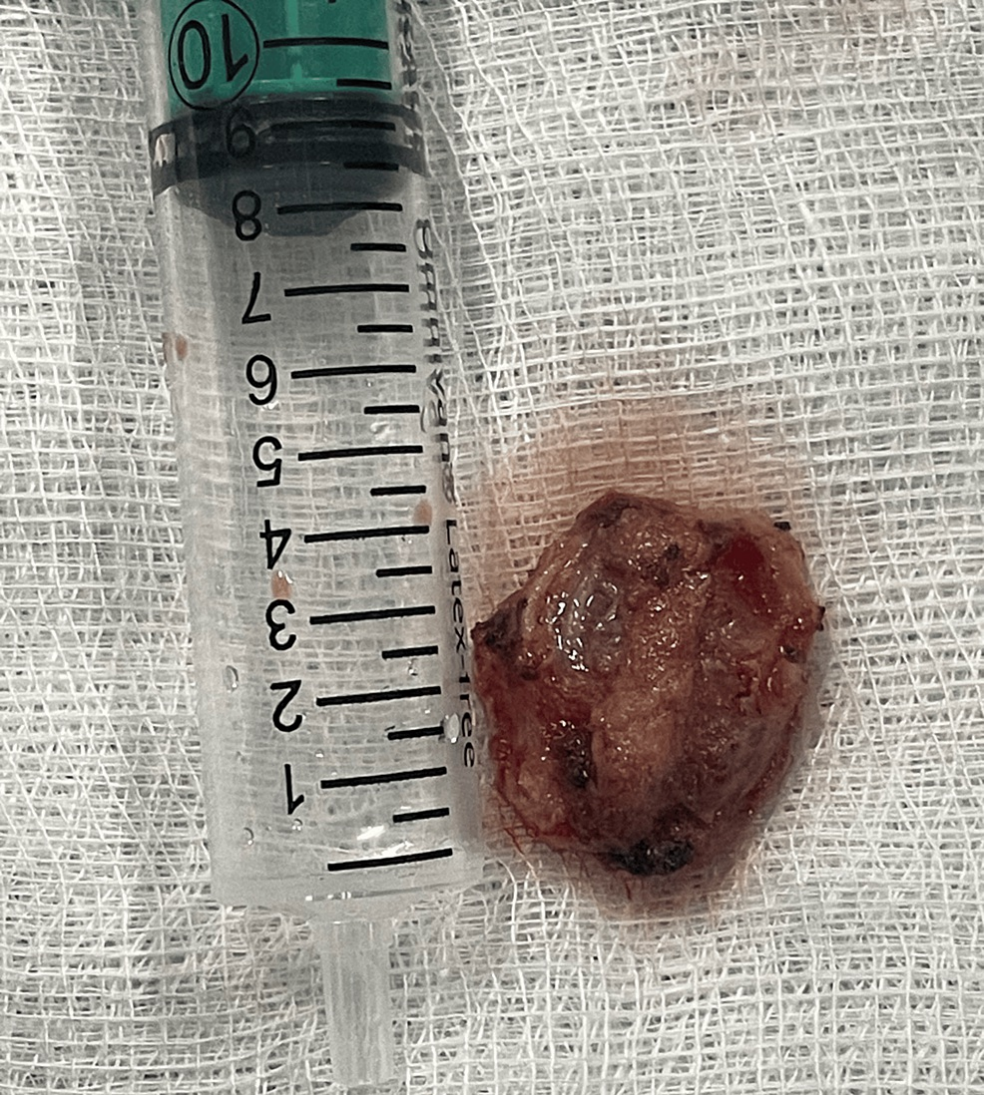
The excised mass.

All routine blood and radiological investigations were done and were within normal limits. The USG Doppler of the upper lip revealed a well-defined hypoechoic lesion showing internal vascularity within which there was arterialized flow with no obvious deeper extension or fat stranding. The following features were suggestive of arteriovenous malformation: After obtaining fitness for surgery by the anesthesiologist and written informed consent from the patient, the patient was taken up for the excision of the upper labial swelling under general anesthesia (Figure 2). Further, the excised mass (Figure 3) was sent for histopathological testing. Hemostasis was achieved, and the postoperative period was uneventful.

**Figure 4:**
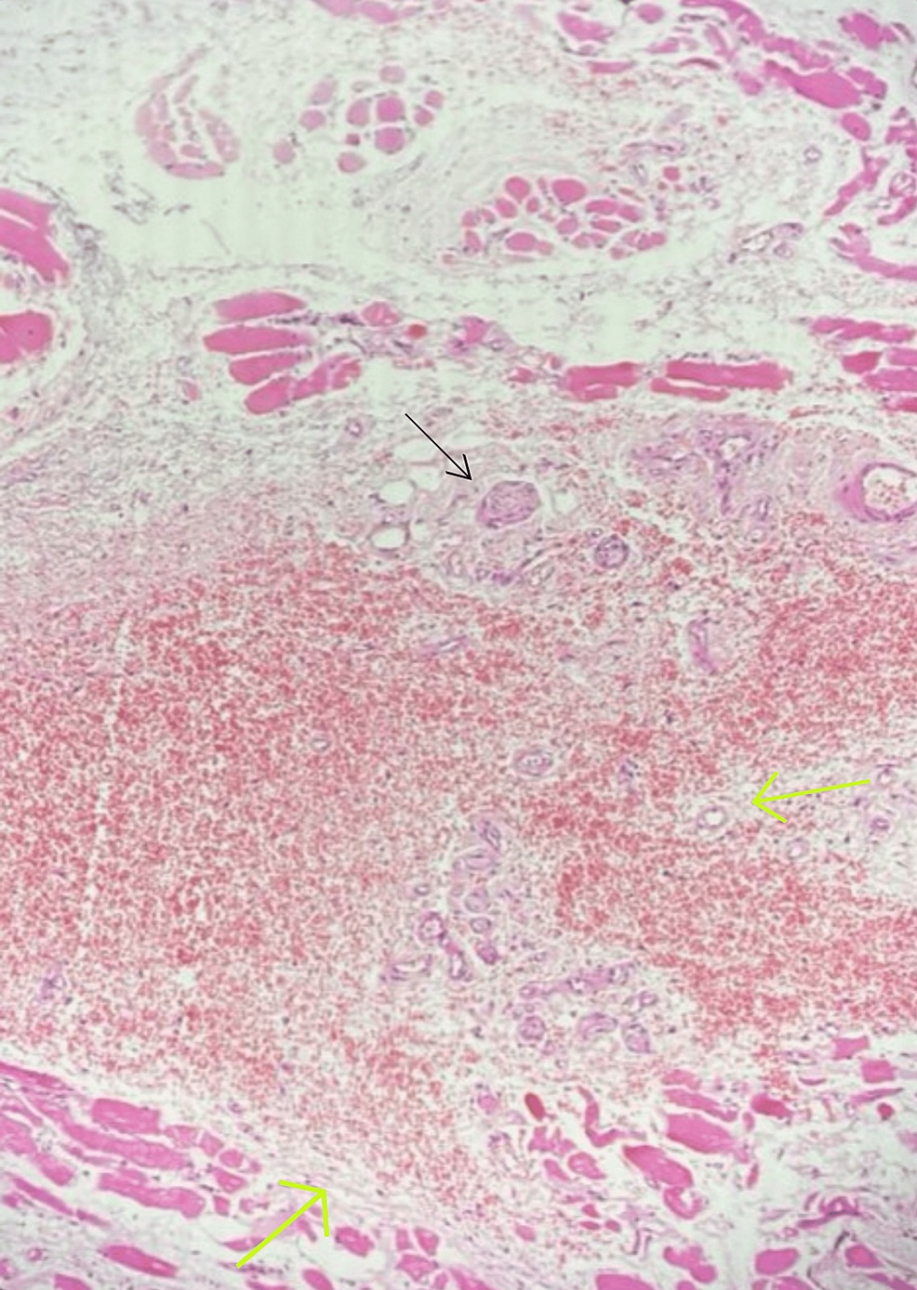
HPE showing stratified squamous epithelium with a circumscribed vascular lesion in the subepithelium consisting of blood vessels with foci of hemorrhages (green arrows). The adjacent mucous glands showing the infiltration of lymphocytes and plasma cells (black arrow). HPE: histopathological examination

The histopathology of the mass (Figure 4) showed stratified squamous epithelium with the subepithelium showing a fairly circumscribed vascular lesion composed of numerous thick- and thin-walled, dilated, congested, and intercommunicating blood vessels with foci of hemorrhages. The adjacent mucous glands reveal infiltration by lymphocytes and plasma cells. There was no evidence of mitosis, necrosis, dysplasia, or malignancy, thus suggestive of arteriovenous malformation.

## Discussion

The abnormalities in vascular development that occur during the fourth and sixth weeks of pregnancy lead to arteriovenous malformations. A deformity might arise from the arterial and venous systems' inability to prune unnecessary, rudimentary connections [4]. The development process of AVMs is yet unknown. These uncommon vascular malformations have been linked to embryologic antecedents, vascular remodeling, and hemodynamic contributions. Trauma, hormones, and previous therapy have all been linked to fast development [3]. Anywhere in the body, including the brain, spinal cord, viscera, bone, skin, and subcutaneous soft tissues, might develop an AVM nidus. The mouth and maxillofacial area have almost half of the lesions, followed by the trunk and extremities. The cheek, nose, ears, and upper lip account for about 70% of maxillofacial AVMs, which are mostly found in the center of the face. AVM in the scalp is also relatively not uncommon [5].

Allison and Kennedy have developed a simpler categorization scheme for peripheral arteriovenous malformations based on angiographic flow parameters, as well as clinical criteria. Three groupings have been recognized: Group 1, which includes lesions primarily arterial or arteriovenous; Group 2, which includes tender vascular lesions, such as capillaries; and Group 3, which includes lesions that are mostly venous [6].

Direct lines of communication between veins and arteries, known as AVMs, eschew the capillary plexus and are present in congenital (faulty transforming growth factor-beta {TGF-β}), familial (mutated *RASA1* gene), and acquired forms. Acquired AVMs often have a single feeder, as opposed to congenital variants, which have many feeding arteries. The clinical phases are as follows: stage 3 is characterized by local damage and ischemia, whereas stage 4 is characterized by high-output cardiac failure [7].

Selective angiography is the primary tool for studying the arterial blood supply. For face AVMs, the Doppler ultrasonic flow detector is employed to detect both internal and exterior carotid flow. Tomograms and CT scans are not very useful for examining these lesions. Despite the lack of pertinent research in the recent literature, digital subtraction angiography shows potential in the study of face AVMs [8].

However, clinical judgment alone may typically distinguish between distinct kinds of lesions without the need for complex radiological or histological examination [9].

AVMs differ in their clinical manifestations, histological characteristics, and results. The diagnosis and treatment of these individuals may entail a large number of specialists from various specializations. The development patterns and hemodynamic characteristics of these masses play a crucial role in deciding the best course of therapy for AVM. The typical ways to treat these lesions involve multimodal therapy such as embolization or preoperative sclerosing drugs [10]. The management of AVMs is debatable. Head and neck AVMs have been managed using a variety of techniques throughout the last 10 years, such as surgical excision, endovascular embolization, laser treatment, or a combination therapy. The most successful treatments for AVMs have been found to involve embolization and excisional surgery. Onyx, gel foam, coils, glue, embosphere, and polyvinyl alcohol are the embolizing agents that are employed [11]. Although complications from embolization are rare, it is possible for nearby tissues to necrotize.

Therefore, broad-spectrum antibiotics must be used to treat patients appropriately. The most dangerous side effect is when the embolus reflows into the vertebral or internal carotid arteries as a result of impaired circulation. This problem can be avoided by putting the microcatheter near to the nidus at a distal location [4].

## Conclusions

In summary, this case emphasizes the difficulties in identifying arteriovenous malformations (AVMs) and stresses the significance of thorough clinical judgment, angiography, and histological evaluation. With an emphasis on embolization and excisional surgery, successful surgical resection highlights the efficacy of multimodal therapy in the management of such intricate vascular abnormalities. In order to provide individualized therapies for the best possible patient outcomes, the multidisciplinary approach is still essential for negotiating the complexities of AVMs.
